# Laparoscopic Inguinal Hernioplasty with a Polyether Ether Ketone Anchoring Device in Intact Male Horses Does Not Compromise Testicular Perfusion, Sperm Production or Motility Characteristics

**DOI:** 10.3390/ani15030402

**Published:** 2025-01-31

**Authors:** Arantza Vitoria, Laura Barrachina, Antonio Romero, Sara Fuente, Ignacio de Blas, Lydia Gil, Francisco José Vázquez

**Affiliations:** 1 Servicio de Cirugía y Medicina Equina, Hospital Veterinario de la Universidad de Zaragoza (HVUZ), C/Miguel Servet 177, 50013 Zaragoza, Spain; avm@unizar.es (A.V.); aromerol@unizar.es (A.R.); sfuente@unizar.es (S.F.); deblas@unizar.es (I.d.B.); 2 Departamento de Patología Animal, Facultad de Veterinaria, Universidad de Zaragoza, 50013 Zaragoza, Spain; lydiagil@unizar.es; 3 Instituto Universitario de Investigación Mixto Agroalimentario de Aragón, Universidad de Zaragoza, 50013 Zaragoza, Spain; 4 Departamento de Anatomía, Embriología y Genética Animal, Facultad de Veterinaria, Universidad de Zaragoza, 50013 Zaragoza, Spain

**Keywords:** horse, stallion, inguinal hernia, hernioplasty, laparoscopy, PEEK harpoon, testicular blood flow, Doppler ultrasound, sperm production, seminal characteristics

## Abstract

A new surgical technique using a polyether ether ketone (PEEK) anchoring device for testicle-sparing laparoscopic inguinal hernioplasty in stallions was described in 2023 and is known as the PEEK harpoon technique (PHT). In breeding stallions, it is essential that the surgery is effective in preventing inguinal hernia but also that it does not impair the testicular function. This study aims to evaluate whether the PHT may affect testicular function. To achieve that, changes in the testicular blood flow, sperm production and motility characteristics were assessed 28 days after use of the PHT. Standing laparoscopic hernioplasty using the PHT was performed unilaterally in eight healthy (non-previously herniated) experimental intact males. The contralateral inguinal canals and testicles were used as control. Pre- and post-surgery Doppler ultrasonographic evaluations of testicular perfusion were performed serially. Bilateral castration was performed at 28 days post-surgery, and epididymal sperm were obtained from both testicles to analyze seminal characteristics. No significant differences were identified in regard to testicular perfusion and the sperm characteristics of the control and operated testicles, suggesting that the PHT-based hernioplasty would not compromise testicular function. Further studies evaluating effects over a longer period are needed, but our data indicate that the PHT is suitable for testicle-sparing inguinal laparoscopic hernioplasties in breeding stallions with a history or predisposing factors of inguinal herniation.

## Simple Summary

In stallions and intact male horses, a segment of intestine can accidentally enter into the inguinal canal that connects the external (scrotal) testicles with the abdominal cavity, resulting in an inguinal hernia. These hernias often are life-threatening, so different techniques have been developed to prevent them. Laparoscopy is a minimally invasive surgical technique that allows for partial closure of the inguinal canal, allowing the passing of vessels but preventing the passing of bowels. For this purpose, a new surgical technique using an anchoring system based on a biocompatible harpoon was developed. However, it also needs to be evaluated whether this partial closure of the canal does not affect the testicular blood flow and sperm production. This surgery was performed only in one inguinal canal of eight healthy experimental intact males. The contralateral testicles were used as control. Pre- and post-surgery evaluation of the testicular blood flow was performed. After 28 days, bilateral castrations were carried out and the sperm of each testicle was separately obtained to analyze seminal characteristics. No abnormalities were detected. Longer-term studies are needed, but our data suggest that this new technique could be safely used in stallions to prevent herniation while keeping their reproductive value.

## 1. Introduction

Inguinal herniation is a serious condition in stallions. This condition can be life-threatening and, even when successfully resolved, can compromise the reproductive use of the stallion, since full closure of the inguinal canal (IC), and thus the removal of the affected testicle, are often required. Therefore, IC partial closure, i.e., inguinal hernioplasty (IH), can be an important prophylactic measure in stallions with predisposing factors or with a history of inguinal herniation [1,2]. The IH can be performed laparoscopically in the standing horse, and several techniques have been developed to serve this purpose. Among others, mesh insertion within the IC [3,4], peritoneal flap [5,6], vaginal ring suture [7,8], cyanoacrylate glue injection into the IC [9], tacked intraperitoneal slitted mesh (TISM) [10], and barbed sutures [11] have been reported. However, some of these techniques usually require advanced laparoscopic skills or do not include a large amount of tissue to ensure strong seals in cases with very large vaginal rings.

Our group recently reported the use of a polyether ether ketone (PEEK) anchoring device for laparoscopic testicle-sparing IH in intact male horses [12]. This technique uses a PEEK harpoon that is anchored in the craniolateral aspect of the vaginal ring via a special applicator and fixed using an extracorporeal knot. Briefly, the harpoon is mounted on the applicator within its protective sheath, directed to the craniolateral aspect of the vaginal ring (approximately 1 cm below its dorsal edge), and anchored into the musculature of the parietal part of the vaginal ring. The harpoon has attached two ends of a polyvinylidene fluoride (PVDF)-threaded suture, through which a button is passed and an extracorporeal knot is used to fix it against the vaginal ring.

This previous work focused on preliminarily assessing the feasibility of the PEEK harpoon technique (PHT), suggesting that it could allow for a simpler and faster system for laparoscopic IH in stallions. Serial clinical and lameness examinations, as well as a laparoscopic re-evaluation, suggested that the PHT was safe in the short term [12]; however, testicular function was not assessed in that study. Provided that one of the main goals of testicle-sparing IH techniques is to safeguard the reproductive value of the stallion, it is crucial to determine whether the PHT affects testicular function. Sperm production, both in regard to number and quality, is the most obvious reflection of testicular function [13]. However, provided that the IC is partially closed and, thus, the spermatic cord (*funiculus spermaticus*) can be compressed, assessing testicular blood flow is also of great importance to ensure an adequate blood supply [14].

The current study is a continuation of our previous work to assess the testicular function in the same animals on which the PHT was performed. We aimed at extending the information on the safety profile of this new IH technique, assessing testicular blood flow and sperm production after partially closing the IC with the PEEK harpoon anchoring system. Our hypothesis was that there would not be significant differences between operated and non-operated (control) testicles in terms of blood flow (serial assessment) and sperm production (assessed post-orchiectomy).

## 2. Materials and Methods

### 2.1. Study Design

A standing laparoscopic hernioplasty using the PHT was performed unilaterally in healthy (non-previously herniated) experimental intact male horses. The contralateral inguinal canal and testicle were used as control. A pre- and post-surgery Doppler ultrasonographic evaluation of testicular perfusion was performed serially. Bilateral castration was performed at 28 days post-surgery, and epididymal sperm were obtained from both testicles to analyze seminal characteristics.

### 2.2. Animals

Eight experimental intact males (five Spanish Pure Breed [PRE] and three crossbred) were used without signs or a history of inguinal hernia or testicular abnormalities. The age and body weight of the animals were 2–20 years and 400–440 kg, respectively. All the animals were healthy as per the normal limits parameters in the pre-operative clinical examinations. These animals were the same used in the previous study that reported on the PHT for the first time. All procedures were carried out under Project License PI42/14/ approved by the Advisory Ethics Committee for Animal Experiments (CEAEA) from the University of Zaragoza. The care and use of animals were performed accordingly with the Spanish Policy for Animal Protection RD53/2013, which meets the European Union Directive 2010/63 on protecting animals used for experimental and other scientific purposes.

### 2.3. PEEK Harpoon Laparoscopy

One inguinal canal (IC) of each horse was randomly selected to undergo unilateral laparoscopic inguinal hernioplasty (IH), and the contralateral IC and testicle were used as controls. The first four authors and the last one participated in the surgical procedures and thus were aware of which side was operated on in each animal. A standing laparoscopic IH was performed unilaterally on the randomly assigned side following the methodology previously described. Briefly, a PEEK harpoon with a PVDF-threaded suture was anchored in the craniolateral aspect of the vaginal ring. The suture was threaded through a PEEK button that was introduced and directed against the IC. Four extracorporeal knots were used to fix the device. The procedures were performed in the standing horses using a standard protocol of sedation, analgesia and local anesthesia. All animals were operated on by the same surgical team [12].

### 2.4. Post-Operative Assessment of Testicular Function

#### 2.4.1. Testicular Ultrasonography

A basic clinical examination was performed every day of the 28 days of the study. Both testicles were examined by inspection, palpation, B-mode, and color Doppler ultrasonographic evaluation of the testicular perfusion on days 0 (pre-laparoscopy), 1, 2, 3, 7, 21, and 28. Doppler measurements were obtained at the marginal aspect of the testicular artery following the procedure previously described [15]. The blood flow measurements that were recorded were peak systolic velocity (PSV, cm/s), end-diastolic velocity (EDV, cm/s), resistive index (RI), and pulsatility index (PI). These measurements were obtained with the horse restrained in stocks, without sedation, using an ATL Philips (Eindhoven, The Netherlands) equipped with a linear probe (5–12 MHz) (L12-5 38 mm for HDI-3500). In all horses, three values were recorded for each parameter at each time point. These determinations were always carried out by the same operator (first author) on all horses.

#### 2.4.2. Comparison of Epididymal Sperm Characteristics

On day 28, after repeating the laparoscopic exploration, as described in the previous study [12], all horses were subjected to a bilateral standing orchiectomy using an open technique [2]. The castration procedure was performed in the standing horses under the same sedation protocol used for laparoscopy immediately before. In addition, local analgesia was provided by lidocaine 2% injection in the incision site at the scrotum and in the testicular cord. The testicles were individually identified with a code, ensuring blind assessment, and immediately transferred to the reproduction lab to freshly obtain epididymal sperm, separately from each testicle, using a previously described method [16]. To compare the results of the operated testicle versus the control testicle from each animal, the epididymal semen from each testicle was studied separately and not in a pooled ejaculate. The extraction of epididymal contents was performed immediately after orchidectomy, dissecting the epididymis and placing a cannula in the *cauda epididymides*. Subsequently, the epididymis was sectioned at the junction between the body and the tail of the epididymis, and a retrograde washing of the epididymal tail was performed, using in all cases 20 mL of a commercial diluent (INRA 96^®^, IMV Technologies, L’Aigle, France).

The spermatozoa concentration (SC), percentage of live spermatozoa (LIVES), motility and kinematics were an evaluated by Integrated Sperm Analysis System (ISAS^®^ Proiser España), showing the static spermatozoa (SS), motile nonprogressive spermatozoa (MNPS) and motile progressive spermatozoa (MPS). The total sperm parameters were analyzed, with the samples and material being tempered at 37 °C. The parameters established for the analysis were 25 consecutive digitalized images per second, and the particle area was 4–75 μm^2^. With regard to the setting parameters for the sperm, the values of Average Path Velocity (VAP) < 10 μ/s were considered slow, and those >90 μ/s were considered to be fast. Spermatozoa with 75% of the straightness index (STR) were designated as progressive motile sperm. In addition, the hypo-osmotic swelling (HOS) test [17] was performed to assess the spermatozoa membrane integrity. Finally, the percentage of normal acrosomes was evaluated using the technique described by Pursel (ACROSOM) [18]. All seminal determinations were blindly analyzed by the fifth author.

### 2.5. Data Analysis

A data analysis was performed by commercial statistical software (IBM SPSS Statistics for Windows, version 19.0, released 2010). The normal distribution of quantitative variables was checked with the Shapiro–Wilk test, and variables were described according to their arithmetic mean and standard deviation (SD) for normally distributed variables and using median and interquartile range (IQR) for non-normal variables.

For the analysis of Doppler ultrasonography parameters (PSV, EDV, RI, PI), a repeated measures ANOVA was applied using group (operated, control) and ultrasonographic measurements (3 measures per parameter) as intra-subject factors and stratifying by day post-intervention.

Regarding the statistical analysis of the sperm characterization (SC, SS, MNPS, MPS, LIVES, HOS, ACROSOM), only one time point was assessed so control vs. operated testicles were compared using a paired Student’s *t*-test for dependent samples (for normally distributed variables) or the Wilcoxon’s test (for non-normally distributed variables).

For all the tests, the *p*-value was obtained, and significance (error α) was set at 0.05 in all cases.

## 3. Results

The eight animals used in the prospective study recovered successfully from the surgical procedure with no remarkable intraoperative complications. No abnormalities were observed upon basic clinical examination, testicle examination and palpation or B-mode ultrasonography within 28 days after surgery.

### 3.1. Testicular Perfusion

The results of the Doppler evaluation of the testicular perfusion are shown in Table 1. No statistically significant differences between control and operated (treated) testicles were identified at any time point for any parameter. All values were within the reference ranges previously reported [2].

### 3.2. Orchidectomy and Sperm Analysis

All horses were castrated with no incidences and with no significant complications during surgery or post-operatively. Differences between both testes of each animal (i.e., one operated, one control) were not noted during the orchiectomy.

All the sperm values were within the reference range as defined [19,20,21,22] in epididymal semen. No statistically significant differences were observed between the sperm sample obtained after epididymal collection of both the operated and control testes in regard to the total sperm count, sperm motility and seminal endosmosis test, nor were differences observed in the percentage of normal acrosomes and in the percentage of live and dead spermatozoa (Table 2).

## 4. Discussion

Inguinal hernioplasty techniques with testicular preservation in breeding stallions require the study of the influence of the surgical technique on testicles. Some works have shown complications after performing IH with different techniques, such as some cases of scrotal oedema [3] and transient haemospermia [6]. The potential impact of hernioplasty on the reproductive functionality of stallions is an important aspect in assessing the safety of these techniques. However, these evaluations have not been performed comprehensively in any of the published studies of laparoscopic hernioplasty with clinical cases [23]. In general, these studies do not provide specific data on fertility but only mention the preservation of reproductive function or the absence of a negative impact on it [5,6,9,10,11]. These aspects have been studied in more detail in experimental animal studies that analyzed the impact of some laparoscopic hernioplasty techniques on spermiograms [13] and testicular perfusion [14]. In human medicine, the influence of different laparoscopic IH techniques like totally extraperitoneal (TEP) repair and Lichtenstein repair has been evaluated on testicular perfusion and volume, as well as on male fertility. Some human studies have found that techniques using meshes produce transient alterations in testicular vascular RI immediately after surgery, although, in most cases, values return to normal in the long-term follow-up [24,25]. It has also been documented that the use of polypropylene mesh can generate inflammatory reactions and fibrosis, which, in specific cases, have led to complications such as obstructive azoospermia, especially in bilateral procedures [24,25,26]. In contrast, other studies have demonstrated that mesh techniques and hernia repair in boys do not affect testicular perfusion and volume during short- and long-term follow-up [27,28].

Regarding the impact of inguinal hernioplasty on the stallion’s reproductive function, the effect on testicular vascularization is one of the most important parameters. Excessive occlusion of the internal or external inguinal ring can obstruct the testicular artery or veins, leading to ischemic orchitis and, consequently, to testicular atrophy [29]. The PEEK harpoon technique involves placing the harpoon approximately 1 cm below the dorsal edge of the internal inguinal ring (VR), as close as possible to the spermatic cord. However, it should be noted that, if the device is placed much more ventrally, it might affect and damage the spermatic cord. It should therefore be noted that small variations in the technique and in the exact placement of the harpoon could have important repercussions on the parameters studied, which reinforces the need for precision in implant placement. This makes it essential to study the potential impact of this technique on testicular function. The possible effect of laparoscopic IH techniques on testicular vascularization, as well as on sperm production and function, has been studied mainly in the case of the peritoneal flap technique, which was reported to alter testicular perfusion in horses [14] without affecting sperm production [13]. In the first study, the PSV of the testicular artery (at the spermatic cord level) was increased after 12 months compared to basal (pre-surgery) values. However, at that time, PI and RI in the marginal portion of the testicular artery were decreased over pre-surgery values [14]. The authors of that study related this pattern of testicular blood flow to a process of testicular hyperemia, and they suggested that the peritoneal flap technique may have compressed the spermatic cord, causing a slight obstruction of the testicular artery that eventually led to compensatory hyperemia to counteract the deficit in the blood flow supply to the testis

In comparison with these works about the peritoneal flap technique, our preliminary results highlighted the safety of the laparoscopic herniorrhaphy technique using a PEEK harpoon, which showed no significant alterations in testicular perfusion parameters in the early post-operative period and at the mid-term follow-up. This suggests that the technique could minimize the risks associated with the use of more reactive materials such as traditional meshes, although further studies with larger samples will be necessary to confirm these findings.

Our results on the assessment of epididymal semen characteristics after 28 days also showed that both semen characteristics and sperm production were not altered by the surgical technique. In our work, in order to be able to evaluate separately the sperm production of the operated and the control testicles, the epididymal content was separately collected after castration. The function of the epididymal head and body regions is considered to be sperm maturation per se, whereas the tail region is thought to be primarily involved in sperm storage prior to ejaculation. In the tail, the specific luminal environment allows spermatozoa to survive for several weeks [30], mainly by maintaining metabolic quiescence and preventing premature sperm activation. During the storage period, the epididymal tail accumulate spermatozoa to ensure that an appropriate amount is available at the time of ejaculation. In bulls and stallions, the amount of stored sperm is approximately sufficient for 10 ejaculations [31].

A potential limitation of this study is the short time after the surgery for castration and epididymal semen collection, which occurred at 28 days post-surgery. Given that equine spermatozoa can survive in the epididymis for several weeks [31], it cannot be discarded that some spermatozoa present in the sperm sample at the time of collection were generated prior to the surgical intervention. Thus, the potential presence of pre-surgery spermatozoa in sperm analyses should be considered. Nevertheless, the sperm characteristics were within normal ranges [19,20,21,22] in both the operated and control testicles, with no significant occurring differences between them.

Another limitation of our study was the use of healthy animals with normal-sized vaginal rings and with no history of inguinal herniation or testicular abnormalities. In addition, there could be anatomical differences between a healthy inguinal ring and one with which a hernia has been previously developed, and this could influence the success of the surgical procedure and also the possible impact on testicular vascularization or functionality. We chose to use experimental healthy animals for a first evaluation of the safety and usefulness of the technique prior to using it on clinical cases, similarly to previous related works [5]. Therefore, it would be very interesting to continue this work in clinical cases, which would also allow us to extend the evaluation of testicular perfusion and sperm production in the longer term and even to check the fertility of reproductive animals by the production of offspring.

It would have also been very interesting to conduct a histological evaluation of the excised testes in our study in order to more accurately assess the effect of the technique on the testes, as other authors have performed previously [4], but, unfortunately, this could not be undertaken in our study.

## 5. Conclusions

In conclusion, the results of our study suggest that the PEEK harpoon technique does not affect either testicular vascularization or sperm characteristics at 28 days post-surgery, thus preliminarily supporting the feasibility of this technique in reproductive stallions. Nevertheless, longer-term follow-up processes with a larger number of stallions, including, if possible, fertility data before and after IH, would be desirable to comprehensively assess the potential effects of the PEEK technique on reproductive functionality.

## Figures and Tables

**Table 1:** Differences in the color Doppler measurements of testicular perfusion at the marginal portion of the testicular artery between operated (*n* = 8) and control (*n* = 8) testicles pre-operatively (day 0) and post-operatively (days 1, 2, 3, 7, 21 and 28) using a repeated measures ANOVA. The measurements were peak systolic volume (PSV), end-diastolic volume (EDV), resistive index (RI) and pulsatile index (PI); data are expressed as mean ± SD. Statistical significance was set for *p* value (*p*) < 0.05.

	PSV			EDV			RI			PI		
Day	Control	Treated	*p*	Control	Treated	*p*	Control	Treated	*p*	Control	Treated	*p*
0	13.358 ± 5.011	11.317 ± 2.547	0.338	4.763 ± 1.717	4.596 ± 1.318	0.831	0.616 ± 0.153	0.584 ± 0.138	0.675	0.920 ± 0.283	0.856 ± 0.247	0.634
1	11.988 ± 3.331	11.521 ± 4.001	0.810	4.700 ± 1.445	4.071 ± 1.307	0.351	0.623 ± 0.111	0.614 ± 0.152	0.893	0.874 ± 0.271	0.913 ± 0.303	0.777
2	10.175 ± 3.340	11.400 ± 3.814	0.511	3.579 ± 1.419	3.958 ± 1.474	0.603	0.635 ± 0.123	0.643 ± 0.116	0.902	0.951 ± 0.238	0.966 ± 0.232	0.901
3	10.163 ± 3.991	12.033 ± 3.818	0.369	3.379 ± 1.588	3.987 ± 1.397	0.437	0.654 ± 0.123	0.648 ± 0.155	0.931	0.996 ± 0.257	0.988 ± 0.339	0.951
7	11.096 ± 3.773	9.029 ± 1.602	0.188	3.842 ± 1.032	3.145 ± 1.208	0.244	0.629 ± 0.121	0.649 ± 0.118	0.746	0.940 ± 0.246	0.981 ± 0.258	0.749
21	11.058 ± 4.068	13.458 ± 3.192	0.224	3.550 ± 1.339	4.554 ± 1.852	0.242	0.633 ± 0.182	0.662 ± 0.101	0.697	0.999 ± 0.323	1.022 ± 0.261	0.871
28	11.508 ± 4.279	11.308 ± 2.047	0.909	3.513 ± 1.213	3.629 ± 1.104	0.842	0.679 ± 0.117	0.670 ± 0.121	0.879	1.048 ± 0.234	1.029 ± 0.255	0.879

**Table 2:** Comparison of spermatic parameters between operated (*n =* 8) and control (*n =* 8) testicles. SC, spermatozoa concentration (×10^6^/mL); SS, percentage of non-motile (static) spermatozoa; MNPS, percentage of motile nonprogressive spermatozoa, MPS, percentage of motile spermatozoa; LIVES, percentage of live spermatozoa; HOS test, percentage of spermatozoa with normal membrane integrity; ACROSOM: percentage of intact acrosomes. Data are presented as median and IQR (interquartile range) for SC with non-normally distributed data (non-parametric Wilcoxon test, W, was used) or as mean ± SD for the other parameters with normally distributed data (paired Student’s *t*-test, t, was used). A statistical significance was set for *p* value (*p*) < 0.05.

	SC	SS	MNPS	MPS	LIVES	HOS	ACROSOM
Control	541.18 (297.70–1410.26)	13.77 ± 8.10	55.34 ± 19.80	31.28 ± 15.96	79.88 ± 9.96	77.13 ± 9.41	74.00 ± 4.81
Treated	618.85 (323.58–1976.83)	15.63 ± 8.56	55.81 ± 9.61	28.55 ± 9.12	82.5 ± 9.00	81.00 ± 10.81	73.13 ± 7.35
*p*	0.674 ^W^	0.589 ^t^	0.931 ^t^	0.577 ^t^	0.510 ^t^	0.458 ^t^	0.760 ^t^

## Data Availability

The original data presented in the study are included in the article; further inquiries can be directed to the corresponding author.
